# Inhibition of the NF-κB Signaling Pathway Alleviates Pyroptosis in Bladder Epithelial Cells and Neurogenic Bladder Fibrosis

**DOI:** 10.3390/ijms241311160

**Published:** 2023-07-06

**Authors:** Jing Chen, Qi Li, Yifan Hong, Xiazhu Zhou, Chengjun Yu, Xiaomao Tian, Jie Zhao, Chunlan Long, Lianju Shen, Shengde Wu, Guanghui Wei

**Affiliations:** 1 Department of Urology, Children’s Hospital of Chongqing Medical University, Chongqing 400014, China; 2 Chongqing Key Laboratory of Children Urogenital Development and Tissue Engineering, Chongqing 400014, China; 3 Chongqing Key Laboratory of Pediatrics, Chongqing 400014, China; 4 Ministry of Education Key Laboratory of Child Development and Disorders, Chongqing 400014, China; 5 National Clinical Research Center for Child Health and Disorders, Chongqing 400014, China; 6 China International Science and Technology Cooperation Base of Child Development and Critical Disorders, Chongqing 400014, China

**Keywords:** neurogenic bladder, spinal cord injury, NF-κB signaling pathway, pyroptosis, fibrosis

## Abstract

Most children with a neurogenic bladder (NB) have bladder fibrosis, which causes irreversible bladder dysfunction and damage to the upper urinary tract. However, the mechanism of bladder fibrosis remains unclear. This study aimed to investigate the underlying causes of bladder fibrosis. Here, the lumbar 6 (L6) and sacral 1 (S1) spinal nerves of Sprague Dawley rats were severed bilaterally to establish NB models. Using RNA-seq, we discovered that the NF-κB signaling pathway and inflammation were upregulated in spinal cord injury (SCI)-induced bladder fibrosis. Subsequent Western blotting, enzyme-linked immunosorbent assays, immunohistochemical staining, and immunofluorescence staining verified the RNA-seq findings. To further clarify whether the NF-κB signaling pathway and pyroptosis were involved in bladder fibrosis, a TGF-β1-treated urinary epithelial cell line (SV-HUC-1 cells) was used as an in vitro model. Based on the results of RNA-seq, we consistently found that the NF-κB signaling pathway and pyroptosis might play important roles in TGF-β1-treated cells. Further experiments also confirmed the RNA-seq findings in vitro. Moreover, using the NLRP3 inhibitor MCC950 rescued TGF-β1-induced fibrosis, and the NF-κB signaling pathway inhibitor BAY 11-7082 effectively rescued TGF-β1-induced pyroptosis and the deposition of extracellular matrix by SV-HUC-1 cells. In summary, our research demonstrated for the first time that the NF-κB signaling pathway inhibition rescued bladder epithelial cells pyroptosis and fibrosis in neurogenic bladders.

## 1. Introduction

Neurogenic bladder (NB) refers to the progressive bladder or urethral dysfunction caused by neurological disease, which is one of the main causes of renal dysfunction [1]. NB often leads to severe bladder dysfunction, including bladder wall thickening, poor compliance, increased intravesical pressure, vesicoureteral reflux, and eventually progression to end-stage renal disease [2]. Around 80% of people with catastrophic spinal cord injuries (SCI) will develop neurogenic bladders and the numerous side effects that accompany them [3]. Loss of coordinated activation of the detrusor and suppression of the urethral sphincter during voiding are further effects of injury to the suprasacral spinal pathways. Instead, a condition known as detrusor–sphincter dyssynergia causes the detrusor and urethral sphincter to contract simultaneously [4]. This leads to increased intravesical pressure, detrusor hyperreflexia, and collagen deposition, which in turn reduces bladder compliance [5], finally causing a multitude of problems including urinary tract infection, vesicoureteral reflux, and renal function impairment. The main cause of pediatric neurogenic bladder (PNB) is congenital spinal cord and neurodevelopmental dysplasia [6]. As the disease progresses, most PNB patients develop different degrees of bladder fibrosis, which is one of the main causes of bladder dysfunction and upper urinary tract damage [7].

Bladder fibrosis is a pathological change in which excess extracellular matrix (ECM) is deposited in the bladder wall layer and is seen clinically in the chronic inflammatory bladder and lower urinary tract obstructive disease due to various causes [8,9]. Massive deposition of collagen fibers in the interstitial or intramuscular spaces can lead to decreased bladder compliance and dysfunction of the detrusor muscle. Current treatments for NB include antimuscarinic drugs, clean intermittent catheterization (CIC), intravesical botulinum toxin injection, and surgical treatment. These methods can reduce patient symptoms but fail to delay the progression of bladder fibrosis [10]. Lately, neuroanastomosis therapy for PNB has drawn interest from all around the world, but its therapeutic effect is controversial [11]. Therefore, alleviating bladder fibrosis and protecting the upper urinary tract of patients is the focus of PNB research. However, the mechanism underlying the development of bladder fibrosis in NB is not fully understood.

Recent studies have shown that pyroptosis, a new form of programmed cell death induced by caspases, is closely associated with the development and progression of fibrosis in human organs [12]. In addition, it has been found that epithelial cells play an important role in the fibrosis of many tissues. The bladder epithelium is one of the main cells responsible for the synthesis of the extracellular matrix [13]. In our previous study, we discovered that NB rat bladder epithelial cells experience pyroptosis, but whether it plays a role in NB fibrosis is unclear [14]. As a classic pathway, the downstream inflammatory pathway of NF-κB may be involved in regulating the accumulation of ECM and the cell cycle [15], and the NOD-like receptor protein 3 (NLRP3) inflammasome is an important component of the inflammatory pathway, which activates Caspase-1 through the NF-κB signaling pathway, causes cellular pyroptosis, and stimulates the maturation and secretion of proinflammatory cytokines (e.g., interleukins, tumor necrosis factor-α) [16,17]. Once extracellular pattern recognition receptors identify damage- or pathogen-associated molecular patterns, the NF-κB signaling pathway is activated, which causes the transcription of NLRP3, pro-IL-1β, and pro-IL-18 to be increased. Several intracellular triggers then activate NLRP3 and Caspase-1, which causes the GSDMD to mediate the creation of membrane holes. This eventually causes the proinflammatory cytokines IL-1β and IL-18 to mature and release [18]. Therefore, we propose the hypothesis that neurogenic bladder may trigger NF-κB activation, NLRP3 inflammasome expression, and extracellular matrix deposition in bladder epithelial cells under pathological conditions. 

In conclusion, inflammatory stimulation of the neurogenic bladder can exacerbate bladder tissue fibrosis in several ways. Pyroptosis, a mechanism of programmed inflammatory cell death, may contribute to the development of bladder fibrosis after SCI and may be controlled by the NF-κB signaling pathway. In the current investigation, we examined changes in the expression of pyroptosis-related proteins both in vitro and in vivo. We discovered that inhibiting the in vitro expression of the NF-κB signaling pathway prevented pyroptosis of bladder epithelial cells and reduced bladder fibrosis. Our research will shed new light on the mechanisms underlying SCI-induced bladder fibrosis.

## 2. Results

### 2.1. Establishment of the Neurogenic Bladder Fibrosis Model

**Figure 1:**
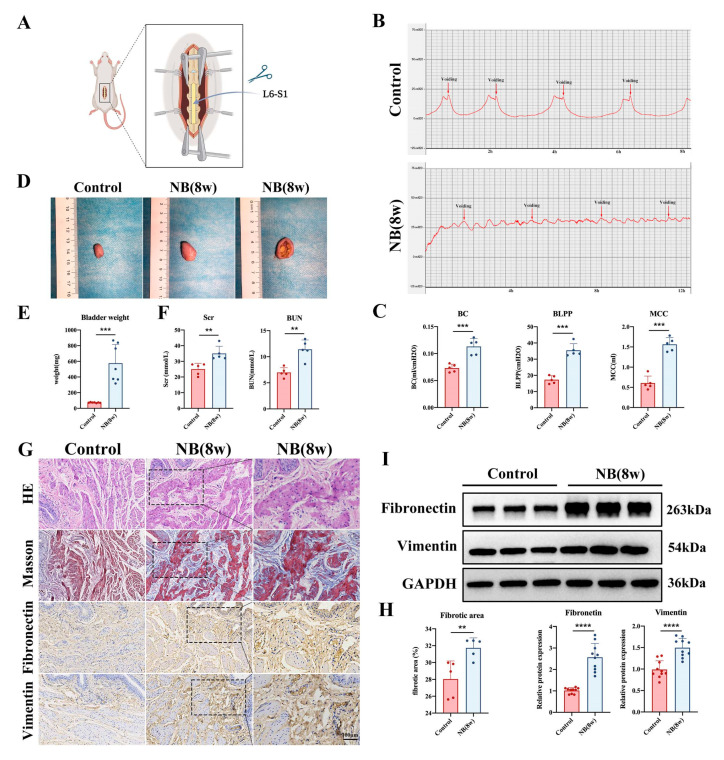
Bladder and upper urinary tract dysfunction and fibrosis in the bladders of rats after SCI. (**A**) The L6-S1 spinal nerves of SD rats were bilaterally severed to build the NB model. (**B**) Cystometric curves of rats. The horizontal coordinate indicates the time of perfusion and the vertical coordinate indicates the intravesical pressure (cmH_2_O). (**C**) Comparison of cystometric parameters (BC, BLPP, MC) in the control and NB groups *(n* = 5 each group). (**D**) Gross morphological changes in bladder tissues in the different groups of rats. (**E**) Weight changes in bladder tissues between the two groups (*n* = 7 each group). (**F**) Scr and BUN levels in the two groups of rats (*n* = 5 each group). (**G**) H&E, Masson, and immunohistochemical staining (scale bar = 100 μm) of bladders in the two groups of rats. (**H**) Based on five randomly chosen fields of stained bladder tissue slices, the fractional area of fibrosis in the bladder was quantified. (**I**) Relative protein levels of fibronectin and vimentin in the two groups were analyzed by Western blotting. The data are presented as the mean ± SD; ** *p* < 0.01, *** *p* < 0.001, **** *p* < 0.0001.

The L6-S1 spinal nerves of Sprague Dawley (SD) rats were bilaterally severed to understand the underlying causes of bladder fibrosis (Figure 1A). We performed urodynamic examinations on rats after SCI and found that rats in the sham-operated group showed substantial peak contraction of the detrusor muscle during the voiding phase. However, the bladders of rats in the SCI group did not have a distinct filling and voiding phase and showed intermittent urinary incontinence (Figure 1B). In addition, there was a great increase in maximum bladder capacity (MCC), bladder leak point pressure (BLPP), and bladder compliance (BC) in the NB group at 8 weeks post-surgery (Figure 1C), indicating severely impaired bladder function. Then, the bladder tissues of rats were collected for morphological observations and weighing. The findings demonstrated that SCI considerably increased the bladder capacity in the NB group and even caused bladder stones to develop (Figure 1D). Moreover, the weight of bladder tissue increased dramatically in the NB group (Figure 1E).

To determine whether impaired bladder function further affected upper urinary tract function, we tested renal function. The results showed that Serum creatinine (Scr) and blood urea nitrogen (BUN) levels in rats were elevated in the NB group, indicating impaired renal function (Figure 1F). Then, we performed histological examinations of bladder tissues. Hematoxylin and eosin (H&E) staining showed that bladder tissues in the NB group had thickened muscle bundles, disorganized arrangement, and obvious inflammatory cell infiltration in the epithelial region. Collagen fibers were colored blue after Masson’s staining, while bladder smooth muscle cells were colored red. Measurements were made of the collagen area to total area ratio. In comparison to the control group, the NB group’s fractional area of bladder fibrosis was considerably higher (Figure 1G,H). Subsequently, the immunohistochemical and Western blotting results showed an increase in fibronectin and vimentin expression in bladder tissue in the NB group, indicating increased fibrosis (Figure 1G,I). Overall, these results suggest that bladder and upper urinary tract dysfunction occurred in rats after SCI and induced fibrosis in the bladder.

### 2.2. The NF-κB Signaling Pathway and Inflammation Were Enriched in Neurogenic Bladder Fibrosis

**Figure 2:**
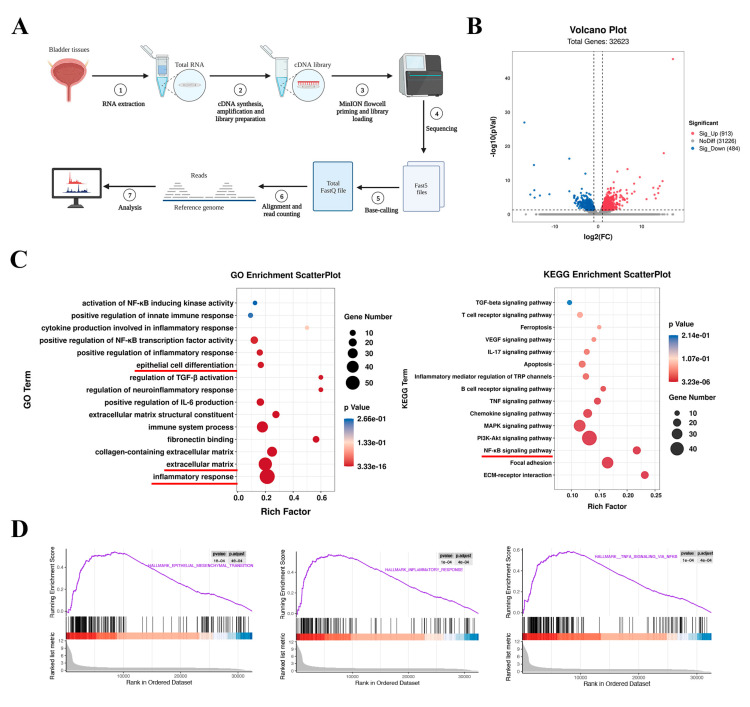
Bioinformatics analysis of the transcriptome sequencing results of tissues. (**A**) Schematic diagram of the RNA-seq and biochemical analysis process. (**B**) Using a volcano plot, we identify the genes that are differentially expressed between the control and NB groups. (**C**) The NF-κB pathway, epithelial cell differentiation, and ECM were shown to be abundant in the DEGs of tissues according to GO and KEGG analyses (the red underline indicates the focus of attention). (**D**) Potential enhancement of EMT, the NF-κB pathway, and the inflammatory response in tissues was examined using GSEA.

To investigate the mechanism of bladder fibrosis in the NB group, we subjected the tissue transcriptome sequencing results to differential analysis and enrichment analysis (Figure 2A). Using comparative analysis, we found 1397 genes that were abnormally expressed in the NB group. A total of 913 of their genes were elevated, whereas 484 were downregulated (Figure 2B). Gene Ontology (GO) and Kyoto Encyclopedia of Genes and Genomes (KEGG) analysis of tissues showed that differentially expressed genes (DEGs) were associated with epithelial cell differentiation, ECM, the NF-κB signaling pathway, and inflammation (Figure 2C). The GSEA analysis also revealed that DEGs were considerably enriched in the NF-κB signaling pathway, inflammation, and EMT (Figure 2D). These results suggest that bladder fibrosis may be related to the NF-κB signaling pathway and inflammatory response.

### 2.3. The NF-κB Signaling Pathway and Pyroptosis-Related Proteins Were Significantly Increased In Vivo 

**Figure 3:**
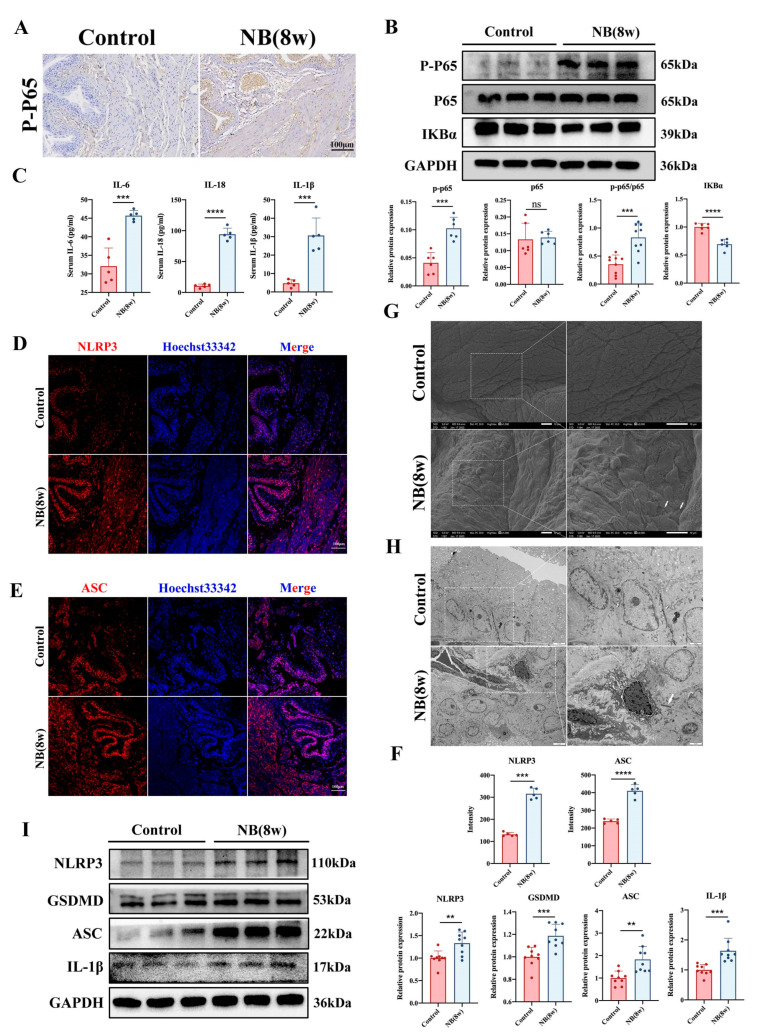
The NF-κB signaling pathway and pyroptosis in rat bladder tissues after SCI. (**A**) Immunohistochemical staining of bladder tissue showed increased expression of P-P65 in the NB group (scale bar = 100 μm). (**B**) The ratio of the NF-κB pathway protein P-P65/P65 and IKBα expression levels in tissues were detected by Western blotting. (**C**) Serum levels of IL-6, IL-1β, and IL-18 in rats (*n* = 5 each group). (**D**–**F**) Immunofluorescence staining and mean fluorescence intensity analysis of NLRP3 and ASC in bladder tissues (scale bar = 100 μm). (**G**) SEM showed disorganized cell arrangement in the epithelial layer of the rat bladder after SCI, increased filamentous secretions, and the generation of pyroptotic bodies. Scale bar = 10 μm, arrows indicate pyroptotic bodies. (**H**) TEM showed cell death, abnormal endoplasmic reticulum morphology and structural disruption. Scale bar = 2 μm, arrows indicate abnormal endoplasmic reticulum. (**I**) Protein expression levels of NLRP3, GSDMD, ASC, and IL1-β in the two groups were detected by Western blotting. The data are presented as the mean ± SD; ** *p* < 0.01, *** *p* < 0.001, **** *p* < 0.0001, ns, not significant.

DEGs were enriched in the NF-κB signaling pathway and inflammatory response, according to the RNA-seq analysis. We first concentrated on the NF-κB signaling pathway, since this is a key mediator of NLRP3 inflammasome-mediated pyroptosis [18]. We performed immunohistochemical staining of rat bladder tissue sections and found that the bladder epithelium displayed considerably higher levels of P-P65 expression (Figure 3A). Subsequent Western blotting confirmed the activation of the NF-κB signaling pathway in bladders in the NB group (Figure 3B). The inflammatory factors IL-6, IL-1β, and IL-18 were increased in the serum of rats after SCI compared with those in the control group (Figure 3C). To further investigate whether the inflammatory response of bladder tissue in the NB group was associated with pyroptosis, we subjected bladder tissue to immunofluorescence staining. The results showed an increase in the expression of NLRP3 and ASC, which are proteins associated with pyroptosis, in the bladder epithelium (Figure 3D–F). Subsequently, using scanning electron microscopy (SEM), we observed disorganized cell arrangement, increased filamentous secretion, and the generation of pyroptotic bodies in the bladder epithelial layer in the NB group (Figure 3G). Transmission electron microscopy (TEM) showed cell death and abnormal endoplasmic reticulum morphology in the bladder epithelium in the NB group (Figure 3H). Western blotting later revealed that the bladder tissue of the NB group had higher levels of NLRP3, GSDMD, ASC, and IL-1β expression (Figure 3I). These results indicated that the bladder tissue of rat was structurally disturbed by cell death and the inflammatory response after SCI, which might be related to bladder epithelial cell pyroptosis.

### 2.4. TGF-β1-Induced Fibrosis Model of SV-HUC-1 Cells In Vitro

**Figure 4:**
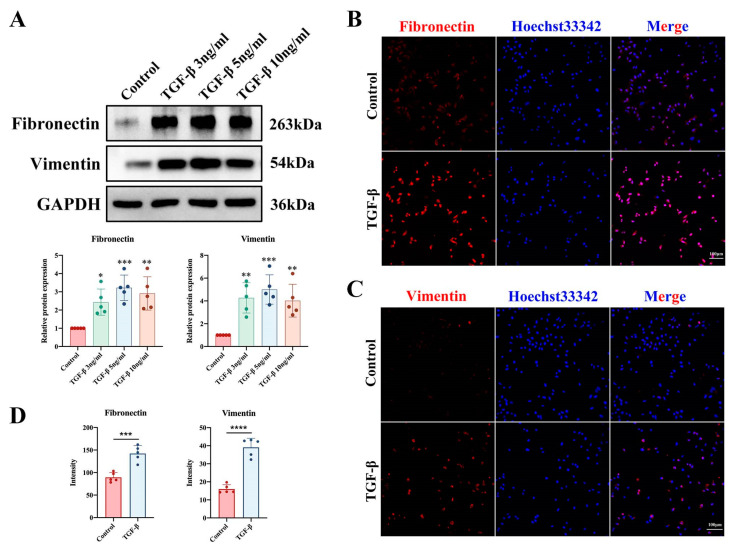
Increased cellular fibrosis after the addition of TGF-β1 to bladder epithelial cells. (**A**) Western blot analysis of the effect of different concentrations (3, 5, and 10 ng/mL) of TGF-β1 on cell fibrosis. (**B**–**D**) Cellular immunofluorescence staining showed changes in the expression levels of the cellular fibrosis markers fibronectin and vimentin in both groups (scale bar = 100 μm). The data are presented as the mean ± SD; * *p* < 0.05, ** *p* < 0.01, *** *p* < 0.001, **** *p* < 0.0001.

To further explore whether the pyroptosis and NF-κB pathway were involved in regulating bladder epithelial cell fibrosis, we further constructed a cellular model of bladder epithelial cell fibrosis. Previous studies have shown that TGF-β1 is a key regulator of the ECM, cell proliferation and differentiation, and the inflammatory response in fibrotic diseases [19]. TGF-β1 is frequently employed in experimental investigations to induce pulmonary fibrosis [20], renal fibrosis [21], and hepatic fibrosis [22] since it is essential for wound healing and the deposition of matrix molecules. Therefore, we used different concentrations (3, 5, and 10 ng/mL) of TGF-β1 to induce ECM in SV-HUC-1 cells. Western blotting was used to detect the protein expression of fibronectin and vimentin, and the results showed that 5 ng/mL TGF-β1 effectively induced ECM in SV-HUC-1 cells (Figure 4A). Based on this outcome, we chose 5 ng/mL TGF-β1 for the subsequent experiments. To further demonstrate that ECM accumulation occurred in TGF-β1-treated cells, we performed immunofluorescence assays and found that the expression of fibronectin and vimentin was increased (Figure 4B–D), indicating that the in vitro cell fibrosis model was established.

### 2.5. The NF-κB Signaling Pathway and Inflammation Were Enriched in TGF-β1-Induced SV-HUC-1 Cells

**Figure 5:**
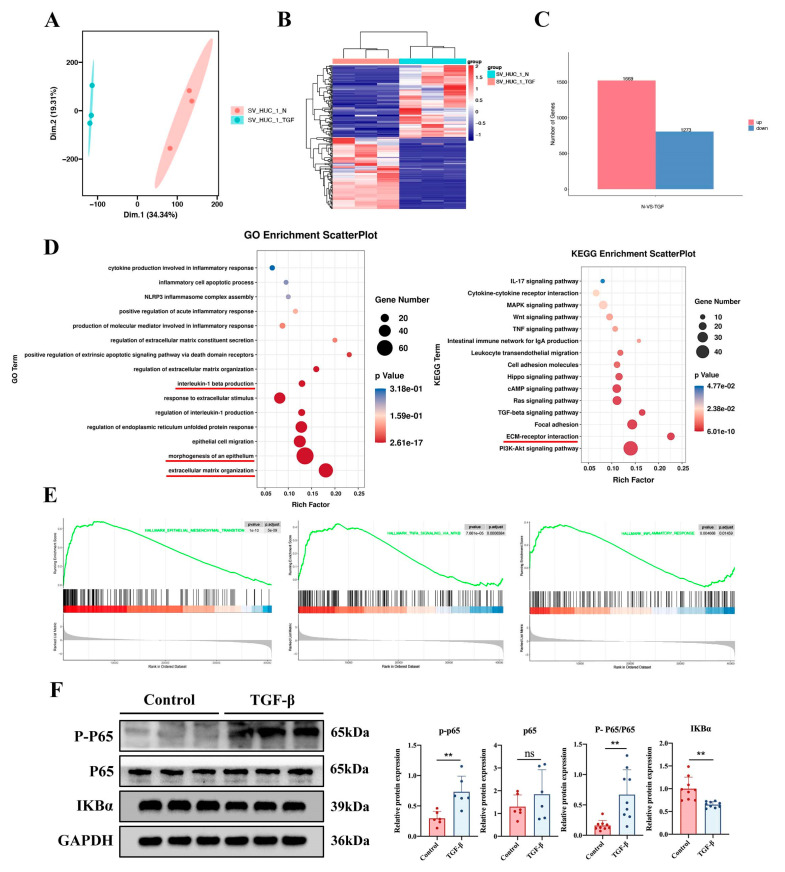
Bioinformatics analysis of the transcriptome sequencing results of cells. (**A**) RNA-seq data from cell samples were subjected to principal component analysis (PCA). (**B**) Cell sample correlation heatmap for the control and TGF-treated groups. (**C**) DEGs counts in the control and TGF-treated groups. (**D**) Cellular DEGs were shown by GO and KEGG analysis to be upregulated in pathways associated with epithelial cell migration, ECM, and the inflammatory response in the TGF-β1-treated group (the red underline indicates the focus of attention). (**E**) Potential enhancement of EMT, the inflammatory response, and the NF-κB pathway in cells was examined using GSEA. (**F**) The ratios of the NF-κB pathway protein P-P65/P65 and IKBα expression in cells treated with TGF-β1 were examined by Western blotting. The data are presented as the mean ± SD; ** *p* < 0.01; ns, not significant.

Transcriptome sequencing was performed on cells from the control and TGF-β1-treated groups (n = 3). As shown in Figure 5A, the samples from the control and TGF-treated groups were evenly distributed across the groups. Moreover, the samples were fully distinct from one another, indicating that each group had the appropriate level of aggregation. Then, a heatmap analysis of cell sample correlation revealed variations between the groups and excellent reproducibility within the sample groups (Figure 5B). About 2942 DEGs were discovered, and the numbers of up- and downregulated genes are shown in Figure 5C. We then performed GO and KEGG analysis of DEGs in both groups of cells, and the results showed that DEGs were upregulated in the pathways associated with epithelial cell migration, ECM, and the inflammatory response in the TGF-β1-treated group (Figure 5D). GSEA revealed that DEGs were considerably enriched in the NF-κB signaling pathways, EMT, and inflammatory response (Figure 5E). To demonstrate the activation of the NF-κB signaling pathway in cells, Western blotting was performed, and the results showed that the P-P65/P65 ratio was enhanced in response to TGF-β1, and the expression of IKBα was decreased (Figure 5F). These results suggest that bladder epithelium cell fibrosis may be related to the NF-κB signaling pathway and inflammatory response.

### 2.6. Pyroptosis-Related Proteins Were Increased in TGF-β1-Induced SV-HUC-1 Cells

**Figure 6:**
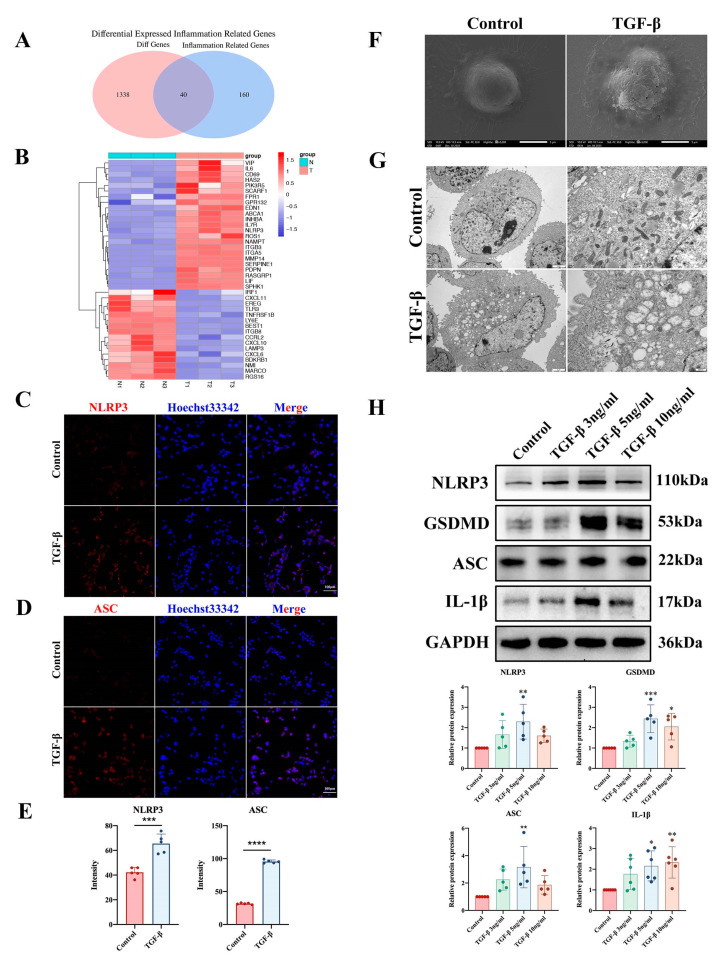
Bioinformatics analysis of the transcriptome sequencing results of cells. (**A**,**B**) Heatmap analysis of IRGs in cellular DEGs. (**C**–**E**) Expression levels of the pyroptosis proteins NLRP3 and ASC in SV-HUC-1 cells were detected by immunofluorescence staining (scale bar = 100 μm). (**F**) SEM showed TGF-β1-induced epithelial cell swelling, cell membrane pore formation, and pyroptotic body generation. Scale bar = 5 μm. (**G**) TEM showed cell membrane disruption, disruption of organelle structures such as mitochondria and the endoplasmic reticulum, and vacuolization. Scale bar = 2 μm and scale bar = 500 nm. (**H**) Protein expression levels of NLRP3, GSDMD, ASC, and IL1-β were detected by Western blotting in vitro. The data are presented as the mean ± SD; * *p* < 0.05, ** *p* < 0.01, *** *p* < 0.001, **** *p* < 0.0001.

To investigate the role of the inflammatory response in bladder fibrosis, we intersected the cellular DEGs with a set of inflammation-related differential genes (IRGs) downloaded from the GSEA website. Finally, we made a heatmap of the differential genes obtained from the intersection and found that most of the inflammatory genes were elevated in cells in the TGF-β1 group (Figure 6A,B). These results suggest that bladder fibrosis may be related to the NF-κB signaling pathway and inflammatory response. Based on the transcriptome sequencing results, we performed immunofluorescence assays of pyroptosis-related proteins in the cells. The results showed that the TGF-β1-induced expression of NLRP3 and ASC was increased in SV-HUC-1 cells (Figure 6C–E). To further confirm pyroptosis in TGF-β1-induced SV-HUC-1 cells, we examined both groups of cells using SEM. We found that the cells were swollen, and cell membrane pores and pyroptotic bodies appeared in the TGF-β1 group (Figure 6F). TEM showed that cell membrane integrity and organelle structures, such as mitochondria and endoplasmic reticulum, were disrupted in the TGF-β1 group (Figure 6G). Western blotting showed that TGF-β1-induced expression of NLRP3, GSDMD, ASC, and IL-1β was increased (Figure 6H). These findings suggest that pyroptosis may be related to bladder epithelium cell fibrosis.

### 2.7. NF-κB Signaling Pathway Inhibition Rescued TGF-β1-Induced Pyroptosis and Fibrosis In Vitro

**Figure 7:**
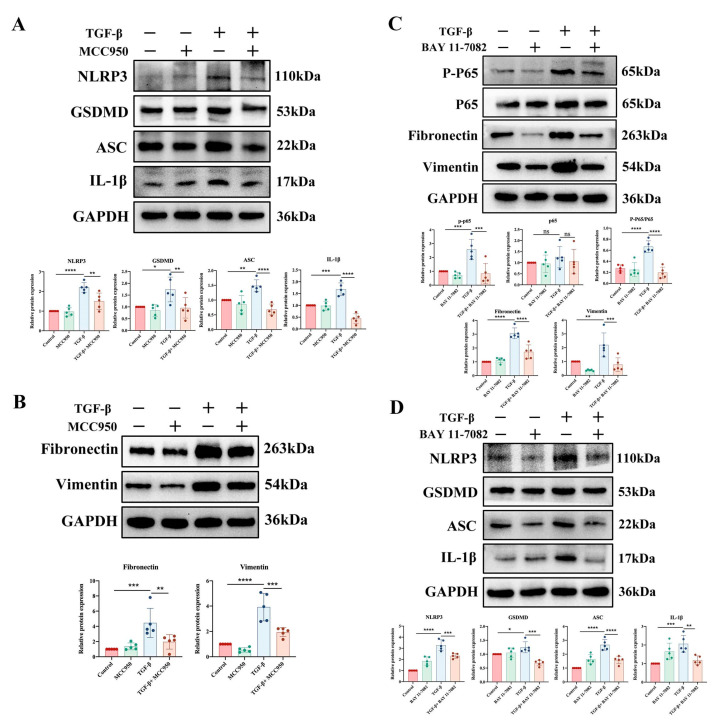
NF-κB signaling pathway inhibition rescued TGF-β1-induced fibrosis and pyroptosis in vitro. (**A**) Protein expression levels of NLRP3, GSDMD, ASC, and IL1-β in cells after the addition of the NLRP3 inhibitor MCC950 were detected by Western blotting. (**B**) Expression levels of fibronectin and vimentin in cells after the addition of the NLRP3 inhibitor MCC950 were detected by Western blotting. (**C**) Western blot analysis of changes in the expression levels of P-P65/P65, fibronectin, and vimentin in cells treated with the NF-κB inhibitor BAY 11-7082. (**D**) Protein expression levels of NLRP3, GSDMD, ASC, and IL1-β in cells treated with the NF-κB inhibitor BAY 11-7082 were detected by Western blotting. The data are presented as the mean ± SD; * *p* < 0.05, ** *p* < 0.01, *** *p* < 0.001, **** *p* < 0.0001, ns, not significant.

To clarify the relationship between pyroptosis and fibrosis, we treated the cells with MCC950 (C*_20_*H*_24_*N*_2_*O*_5_*S), a specific molecular inhibitor of NLRP3. Western blotting showed that MCC950 reduced epithelial cell expression of NLRP3, GSDMD, ASC, and IL-1β (Figure 7A). More importantly, MCC950 reduced the expression levels of fibronectin and vimentin in the TGF-β1 group (Figure 7B). These findings demonstrated that bladder fibrosis might be reduced by decreasing NLRP3 inflammasome-dependent pyroptosis. As the key mediator of NLRP3 inflammasome-mediated pyroptosis, the role of the NF-κB signaling pathway in bladder fibrosis needs further exploration. Subsequently, the elevation of P-P65/P65, fibronectin, and vimentin protein expression levels caused by TGF-β1 was then dramatically reversed after treatment with the NF-κB inhibitor BAY 11-7082 (Figure 7C). To determine the relationship between the NF-κB signaling pathway and pyroptosis, we performed Western blotting and showed that the protein expression levels of NLRP3, GSDMD, ASC, and IL1-β were inhibited by the NF-κB inhibitor BAY 11-7082 (Figure 7D). These findings indicated that the NF-κB signaling pathway promotes NB fibrogenesis via bladder epithelial cell pyroptosis.

## 3. Discussion

PNB is mainly due to progressive bladder dysfunction caused by spinal nerve abnormalities. Therefore, we constructed an animal model of PNB caused by SCI for experimental study. With disease progression, bladder fibrosis leads to severe bladder dysfunction, which in turn impairs the function of the upper urinary tract [23]. However, the underlying mechanisms of bladder fibrosis in NB remain unclear. Therefore, we used RNA-seq to examine the involved signaling pathways. We are the first to demonstrate how a new mechanism involving SCI causes pyroptosis in the rat bladder. In particular, we demonstrated that pyroptosis was involved in the development of SCI-induced bladder fibrosis via the NF-κB signaling pathway. 

With the persistence of stress, a chronic inflammatory state develops in the bladder. This leads to the activation of multiple biosynthetic or biodegradation pathways, which is followed by the initiation of wound repair processes and the deposition of excess ECM proteins in the bladder wall, ultimately leading to bladder fibrosis and dyscrasia [24,25]. According to previous studies, the inflammatory response plays a vital role in the development of fibrosis. The balance of collagen synthesis and degradation is disrupted in pulmonary fibrosis as more ECM accumulates in the tissue [26], and increased collagen fragmentation promotes inflammation [27]. Pyroptosis is a form of programmed cell death that accompanies the inflammatory response and can rapidly cause cell expansion, plasma membrane rupture, and the release of cellular contents and large amounts of inflammatory factors [28]. The nonclassical pyroptosis pathway interacts with the classical pathway through NLRP3, which is the most studied inflammasome [29].

Recent studies have shown that fibrosis in a variety of human organs is associated with the NLRP3 inflammasome. Fibrosis in these disorders is mostly influenced by the interaction of the NF-κB/NLRP3/caspase-1/IL-1 axis and TGF-β signaling pathway [30,31]. Bladder outlet obstruction followed by overactivation of the NLRP3 inflammasome pathway in the bladder is closely associated with bladder inflammation, fibrosis, and dysfunction [32]. After treatment of mouse renal tubular epithelial cells (TECs) with TGF-β, NLRP3 induces EMT in TECs, leading to increased expression of α-SMA and MMP9 [33]. In addition, NLRP3^−/−^ [34] mouse models have reduced levels of liver fibrosis. However, the role of the NLRP3 inflammasome in NB fibrosis is unclear.

To simulate PNB caused by SCI, the bilateral L6 and S1 spinal nerves in SD rats were cut. The ECM, inflammatory response, and NF-κB pathway were then considerably enriched in the RNA-seq results, indicating that these pathways may be involved in bladder fibrosis. To further explore whether the NF-κB pathway and pyroptosis were involved in regulating bladder epithelial cell fibrosis, we further constructed a cellular model of bladder epithelial cell fibrosis. We used 3, 5, and 10 ng/mL TGF-β1 to treat cell cultures for 24 h, which was consistent with the in vivo conditions. Subsequently, we sent the control and TGF-β groups of cells for sequencing, and the results were consistent with the tissue sequencing results. Therefore, we investigated the relationship between fibrosis, the NF-κB pathway, and the inflammatory response. The data in this study demonstrate that NLRP3 inflammasome-dependent pyroptosis leads to bladder fibrosis. This mechanism was later confirmed by the use of MCC950, suggesting that NLRP3 mediates pyroptosis and is a target for the prevention of bladder fibrosis.

The NF-κB pathway has long been considered a typical proinflammatory signaling pathway [35]. Previous studies have shown that activation of the NF-κB signaling pathway initiates NLRP3 in the classical pyroptosis pathway. Further assembly of the NLRP3 inflammasome followed by the cleavage of pro-IL-1β and pro-IL-18 by activated caspase-1 promotes the maturation and release of the inflammatory factors IL-1β and IL-18, thereby amplifying the inflammatory response [36]. However, it is not clear whether the NF-κB pathway plays a role in NB fibrosis. In the present study, we suggested that the NF-κB pathway may be the initial factor in bladder fibrosis induced by SCI and may cause the death of bladder epithelial cells.

We observed an increased ratio of P-P65/P65 and decreased levels of IKBα expression in vivo and in vitro. Immunohistochemical analysis showed that P-P65 was mainly expressed in the bladder epithelium. To demonstrate the role of the NF-κB signaling pathway in NB fibrosis, we treated cells with the NF-κB inhibitor BAY 11-7082 and found that inhibiting the NF-κB pathway rescued TGF-β1-induced bladder epithelial cell pyroptosis and fibrosis. Although previous studies have shown that the NF-κB pathway is associated with fibrosis in multiple organs, no studies have demonstrated a role for the NF-κB pathway in NB fibrosis. These findings demonstrate that the NF-κB pathway promotes NB fibrosis by promoting pyroptosis in bladder epithelial cells and indicate a new therapeutic target.

However, there are some limitations to this study. To demonstrate the relationship between pyroptosis and fibrosis, we added the NLRP3 inflammasome inhibitor MCC950 in vitro, but did not validate our findings in vivo. In bladder fibrosis, lesions in the muscular layer of the bladder are important; however, only the role of bladder epithelium in fibrosis was investigated in this study. When performing urodynamic testing, we increased the bladder perfusion rate in the model group to twice as fast in order to shorten the bladder perfusion time, but this may have affected the observed results. In addition, we used only female rats to facilitate urodynamics, which may cause gender bias.

## 4. Materials and Methods

### 4.1. Animals

The Research Ethics Committee of the Children’s Hospital of Chongqing Medical University approved this experimental animal study (protocol code CHCMU-IACUC20210625002). The NB rat model was established as previously described [9]. Once the rats were given 20% urethane (5 mL/kg) anesthesia via intraperitoneal injection, the dorsal and ventral roots of the bilateral lumbar 6 (L6) and sacral 1 spinal nerves (S1) were severed from a dorsal median incision. In the control group, only the nerve roots were exposed to surgical instruments without transaction or any other manipulation. Following surgery, penicillin was constantly given for three days. In order to collect bladder tissue samples for future research, the rats were anaesthetized after 8 weeks using 20% urethane (5 mL/kg), and each rat’s serum was taken using glass vials for specimen storage, then frozen at −80 °C for long-term storage. Then, all of the rats were killed via cervical dislocation, and we promptly collected their bladders for additional studies.

### 4.2. Experimental Design

In this study, L6-S1 spinal nerve transection and sham surgery on SD rats were compared. Twenty female specific-pathogen-free (SPF) SD rats (120–140 g, 6 weeks) were purchased from the Experimental Animal Center of Chongqing Medical University. All rats were fed under SPF conditions at a temperature of 25 ± 2 °C and a relative humidity of 50 ± 5% using a 12 h artificial light/dark cycle, and the rats had free access to water and food. Twenty female SD rats were placed into two groups at random: a sham-operated control group, and an NB group with L6-S1 spinal nerve transection. Our experiments were conducted using a completely randomized design method.

Every rat was a separate experiment unit because of the intervention experiment. Based on the analysis of PASS 15.0 and the primary data collected for our prior study and pre-experiments, whose set power and were 0.85 and 0.05, the sample size was determined [14]. There were no predetermined inclusion or exclusion criteria that were stated explicitly. No rats or data points were left out of the analysis. For each analysis, the group sample size is noted in the figure legend and text. The rats were randomly assigned, and the parameters were chosen by researchers who were blind to treatment groups. We adhered to the ARRIVE criteria for animal research (Animal Research: Reporting of In Vivo Experiments). Morphology, immunohistochemistry staining, immunofluorescence, and protein expression analysis were listed as the main outcome measures throughout the article. In this study, the choice of the statistical test took into account whether or not the data were normalized and was based on a specific dataset.

### 4.3. Urodynamic Study

A catheter was placed into the bladder after the rats were given an intraperitoneal injection of 20% urethane (5 mL/kg) to induce anesthesia. The other catheter aperture was connected to a peristaltic pump and an acquisition system (RM-6240, Chengdu Instrument Factory, Chengdu, China) using a tee connector for fluid infusion and pressure recording. Urodynamic tests were performed by infusing saline into the bladder at 20 mL/h for the control group and 40 mL/h for the NB group and recording parameters such as MCC, BLPP, and BC. Cystometry was performed every 30 min for each rat three times in a row to ensure consistent bladder activity.

### 4.4. Scr and BUN Analysis

Blood samples were collected after the rats had been anaesthetized with 10% chloralhydrate. The serum was then extracted from the samples by centrifuging them at 400× *g* for 10 min at 4 °C. Afterwards, using an autoanalyzer, Scr and BUN in each group were evaluated (Cobas C701, Roche, Basel, Switzerland).

### 4.5. RNA Sequencing Analysis

For the mRNA sequencing analysis, three replicates of each set of SV-HUC-1 cells were used. Moreover, three replicate rat bladders were gathered for the transcriptome study. An Illumina NovaSeq 6000 platform from LC-Bio was used in part for high-throughput sequencing (Hangzhou, China). Using an FDR 0.05 and Log2FC 1 as screening parameters, DEGs between the control and NB groups were analyzed using R software (R version 4.2.0), and genes with low expression levels were eliminated. For a clustering analysis of the DEGs, the R software heatmap was employed, and the R package ggplot2 was used to produce volcanic plots. Then, using R software, the enrichment of these DEGs was examined according to GO and KEGG. IRGs for inflammation were retrieved from the GSEA website (accessed on 9 February 2023 at http://www.gsea-msigdb.org/gsea/index.jsp). The differentially expressed IRGs were then obtained by taking the intersection of DEGs and IRGs.

### 4.6. Histological Analysis

The bladders were embedded in paraffin after being dried and treated in 4% paraformaldehyde. The samples were then divided into sections that were four micrometers thick. Using traditional staining techniques, Masson trichrome and H&E staining were used in the sections to analyze the histomorphology of the rat bladder [14]. The ImageJ programme (V 1.8.0.112) assessed collagen accumulation (% of total area) in Masson’s stained sections. Those who scored the slide results were unaware of the group assignments.

### 4.7. Cell Culture and Treatment In Vitro

We bought SV-HUC-1 cells from Procell Ltd. (Melbourne, VIC, Australia, CVCL_3798). The cells were grown in 5% CO_2_ at 37 °C with 10% fetal bovine serum (VivaCell, Yerevan, Armenia, C04001500) and 1% penicillin/streptomycin in Dulbecco’s modified Eagle’s medium nutrient mixture F-12 (Gibco, C11330500BT, Grand Island, NY, USA). The culture media were supplemented with TGF-β1 (MCE, Zelienople, PA, USA, HEK293) to induce fibrosis in SV-HUC-1 cells. In certain investigations, 3, 5, and 10 ng/mL of TGF-1 were used to activate the cells for 24 h, proteins were extracted. The pyroptosis inhibitor MCC950 (10 μM, MCE, HY-12815A) was used to inhibit pyroptosis in SV-HUC-1 cells for 2 h before TGF-β1 stimulation [37,38]. NF-κB inhibitor BAY 11-7082 (1 M, MCE, HY-13453) was administered to SV-HUC-1 cells for 2 h prior to TGF-β1 stimulation to investigate the involvement of NF-κB signaling pathway in TGF-β1-induced fibrosis. Before usage, all reagents were dissolved in DMSO (MCE, HY-Y0320). The cell lines tested negative for mycoplasma contamination.

### 4.8. Immunohistochemistry 

Deparaffinized bladder tissue slices were hydrated in xylene and ethanol. After microwave heating to repair antigens in the sectioned tissue, endogenous peroxidase was quenched with 3% H_2_O_2_. The sections were blocked with 0.5% bovine serum albumin (Biofroxx, 4240GR0250) for 1 h, and then the sections were incubated with primary antibodies against fibronectin (Zenbio, Durham, NC, USA, 250073) and vimentin (Zenbio, R22775) overnight at 4 °C. After that, the appropriate secondary antibody (Zenbio, 511203) was added. After 1 h, the sections were counterstained with hematoxylin after being stained with 3,3′-diaminobenzidine (Abcam, Cambridge, UK).

### 4.9. Immunofluorescence Staining

A density of 5 × 10^4^ SV-HUC-1 cells per well was used to cultivate the cells on coverslips in 24 well plates. After being treated with TGF-β1, the cells were permeabilized with 0.1% Triton X-100 (Solarbio, China’s Beijing) and fixed in 4% paraformaldehyde for 20 min. After blocking with BSA, the cells were incubated with primary antibodies (1:200) against fibronectin (Zenbio, 250073), vimentin (Zenbio, R22775), ASC (Zenbio, 340097), and NLRP3 (Zenbio, 381207) at 4 °C overnight. Then, the cell slides were treated with a Cy3-conjugated AffiniPure goat antirabbit secondary antibody (AB_2923041) for one hour on the second day. Finally, the tissues were stained for the nucleus for 30 min using Hoechst 33342 (Beyotime, Haimen, China, C1022).

As previously mentioned, bladder tissue pieces were deparaffinized and hydrated. Primary antibodies against NLRP3 (Zenbio, 381207) and ASC (Zenbio, 340097) were incubated on the sections overnight. The slides were then handled similarly to how immunofluorescence staining is performed. Finally, immunofluorescence pictures were taken using a C2 confocal microscope (Nikon, K10587, Tokyo, Japan).

### 4.10. Western Blotting

SV-HUC-1 cells were treated as described above and bladder tissues kept at −80 °C were lysed using RIPA lysis buffer (Beyotime, P0013B) with 10% protease inhibitor cocktail (MCE, HY-K0010). Measurement of the protein concentration was carried out using a BCA Protein Assay Kit (Beyotime, P0012). For Western blotting, protein samples in equal proportions were run through a 7.5–12.5% SDS–PAGE and then transferred to PVDF membranes. The PVDF membranes were incubated with primary antibodies against Fibronectin (AB_2105691), NLRP3 (AB_2923578), Vimentin (AB_2895079), GSDMD (HUABIO, HA721144), ASC (AB_2921364), IL-1β (AB_308765), P-P65 (Zenbio, 310052), P65 (Zenbio, 380172), IkBα (Zenbio, 380682) and GAPDH (Huabio, HA721131) after being blocked with blot-blocking solution (NCM, Suzhou, China) for 10 min. At a ratio of 1:1000, the primary antibodies were diluted. On the second day, the membranes were incubated with goat antimouse (Zenbio, 511103) and goat antirabbit (Zenbio, 511203) antibodies at room temperature for one hour. Target protein bands were then seen using enhanced chemiluminescence substrate (Everbright Ltd., Suzhou, China), and their quantities were determined using Imaging Lab software 4.6.2 (Bio-Rad, Hercules, CA, USA).

### 4.11. SEM and TEM

Isolated animal tissues and cells were immersed in 3% glutaraldehyde and fixed. The ion-sputter coating was applied after the samples had been dried using gradient ethanol and hexamethyldisilazane. A scanning electron microscope (JSM-IT700HR, Japan) was used to take pictures of the samples. The samples were then dehydrated in a gradient ethanol series, embedded in epoxy resin, and postfixed in 1% osmium tetroxide. Ultrathin slices (60 nm) of the treated tissue and cell samples were cut, and they were then stained with lead citrate and uranyl acetate. The sections were then seen using a transmission electron microscope (JEM-1400FLASH, Japan).

### 4.12. Enzyme-Linked Immunosorbent Assay (ELISA)

To obtain the serum, rat blood samples were centrifuged at 4 °C for 10 min. According to the manufacturer’s instructions, ELISA kits (Elab-science, Wuhan, China) were used to determine the concentrations of IL-6, IL-1, and IL-18 in serum.

### 4.13. Statistical Analysis

Each experiment was run at least three times, and all data are reported as the mean ± standard deviation (SD). The SD is displayed in the error bar. Using the Student’s *t*-test, two groups were compared to one another. Several comparisons were made using a one-way ANOVA. Additionally, experimental data analyses were performed with GraphPad Prism 8.0 (San Diego, CA, USA). A *p* < 0.05 was considered statistically significant.

## 5. Conclusions

**Figure 8:**
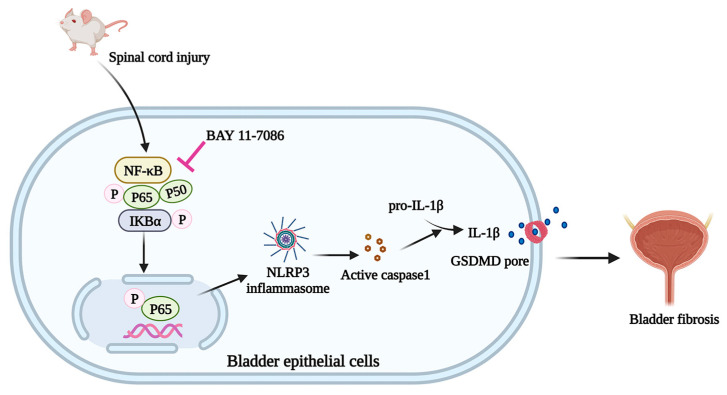
Schematic diagram illustrating that the NF-κB signaling pathway inhibition rescued bladder epithelium pyroptosis and fibrosis in a neurogenic bladder.

In summary, the current study suggests that after severing spinal nerves, the bladder structure was damaged, and function was disturbed. We also showed for the first time that pyroptosis was involved in the development of bladder fibrosis via the NF-κB signaling pathway. Therefore, SCI-induced bladder fibrosis could be ameliorated by inhibiting the activity of the NF-κB/NLRP3 signaling pathway (Figure 8). Our study proposes a strategy for the effective treatment of patients with NB. However, the underlying mechanism still requires further exploration.

## Data Availability

The data presented in this study are available on request from the corresponding author. The data are not publicly available due to privacy.
